# Natural products against tau hyperphosphorylation‐induced aggregates: Potential therapies for Alzheimer's disease

**DOI:** 10.1002/ardp.202400721

**Published:** 2025-01-30

**Authors:** Gustavo Basurto‐Islas, Maximiliano Caye Diaz, Lizeth M. Zavala Ocampo, Melchor Martínez‐Herrera, Perla Y. López‐Camacho

**Affiliations:** ^1^ División de Ciencias e Ingenierías Universidad de Guanajuato León Guanajuato Mexico; ^2^ Departamento de Ciencias Naturales Universidad Autónoma Metropolitana Cuajimalpa Ciudad de México Mexico

**Keywords:** Alzheimer's disease, dementia, plant extract, tau fibrils, tau phosphorylation

## Abstract

Alzheimer's disease (AD) is a progressive neurodegenerative disorder characterized by cognitive decline and memory impairments and is considered the most prevalent form of dementia. Among the contributing factors to AD lies the hyperphosphorylation of the microtubule‐associated protein tau. Phosphorylated tau reduces its affinity for microtubules and triggers other posttranslational modifications that result in its aggregation and assembly into filaments. These structures progressively accumulate within neurons leading to neurodegeneration. While current AD medications often involve undesirable side effects, the exploration of natural products as a potential therapeutic alternative has gained considerable attention. Numerous compounds have shown potential capacity for reducing tau pathology through different mechanisms, such as inhibiting kinases to reduce tau hyperphosphorylation, enhancing phosphatase activity, and blocking fibril formation. Since tau hyperphosphorylation‐induced aggregation is pivotal in AD onset, this review aims to elucidate the potential of natural products in modulating this crucial molecular mechanism.

## Graphical abstract

This review explores the potential of natural products in modulating tau hyperphosphorylation, a pivotal molecular mechanism in Alzheimer's disease onset, by inhibiting kinases, enhancing phosphatase activity, blocking fibril formation, and promoting its disaggregation.

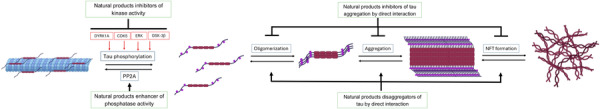

## 1 INTRODUCTION

Alzheimer's disease (AD) is a neurodegenerative disorder and the most prevalent type of dementia. The specific cause of the disease is not yet fully known, but several hypotheses for its pathophysiology have been defined; the most important is focused on the accumulation of both abnormal tau within the cells and amyloid β‐peptide (Aβ) extracellularly.^[^ ^1^ ^]^ These form neurofibrillary tangles (NFTs) and Aβ plaques, respectively. Accumulation of NFTs disrupts mitochondrial regulation and axonal transmission, while accumulation of Aβ plaques induces cellular toxicity, leading to brain damage and cell death. Furthermore, a relationship between these phenomena is proposed.^[^ ^2^, ^3^ ^]^

Currently, AD treatment primarily relies on two categories of medications. The first group treats the symptoms and includes cholinesterase inhibitors (donepezil, rivastigmine, and galantamine),^[^ ^4^, ^5^, ^6^, ^7^, ^8^ ^]^ glutamate regulators (memantine),^[^ ^9^, ^10^ ^]^ and orexin receptor antagonists (suvorexant).^[^ ^11^ ^]^ While all of them have proven effective in treating the symptoms, they have several side effects.^[^ ^12^ ^]^ A more recent development involves a drug that focuses on delaying the clinical decline by targeting and removing Aβ pathology with aducanumab,^[^ ^13^, ^14^ ^]^ but it also shows other complications, such as headache, diarrhea, and upper respiratory tract infection.^[^ ^15^, ^16^ ^]^ Thus, therapeutic approaches focused on reducing Aβ load have failed.^[^ ^17^ ^]^

Furthermore, tau is pivotal in AD onset; therefore, pharmaceuticals that specifically target tau pathology in AD are still in the developmental stage and require further testing.^[^ ^18^ ^]^ As an alternative approach, there is interest in natural products that target tau hyperphosphorylation and its aggregation. These natural compounds offer potential benefits, such as reduced side effects, affordability, and promising effectiveness, which makes them valuable for the AD research community.

## 2 TAU PATHOLOGY IN AD

### 2.1 Tau aggregates

Tau protein is a microtubule‐associated protein (MAP) that stabilizes microtubules in axons, in addition to MAP1 (A/B) and MAP2. It has a ratio of 2–3 mol of phosphate per mol of a molecule, ideal for optimal activity.^[^ ^19^ ^]^ As such, hyperphosphorylation of tau reduces its microtubule assembly and binding activity.^[^ ^20^ ^]^ Hyperphosphorylated tau proteins disassemble from microtubules and aggregate to form paired helical filaments, a double‐helical stack of C‐shaped subunits.^[^ ^21^ ^]^ These polymers ultimately aggregate, forming NFTs and inducing neuronal loss. However, recent studies have proposed that this neuronal loss previously attributed to NFTs is instead due to the unpolymerized abnormal tau or its oligomers.^[^ ^22^ ^]^ Additionally, it has been suggested that NFTs are a marker of previous damage instead of being the cause of such damage.^[^ ^23^ ^]^ Nevertheless, NFTs are the best neuropathological marker of AD's stages.^[^ ^24^, ^25^, ^26^ ^]^

### 2.2 Abnormal tau phosphorylation

In AD, the phosphorylation level of tau is three to four times higher than in normal human brains.^[^ ^27^ ^]^ Besides phosphorylation, tau undergoes other posttranslational modifications, such as acetylation, N‐glycosylation, and truncation. Phosphorylation seems to be the first and most important, with its cleavage and conformational changes following it.^[^ ^28^ ^]^ Tau glycosylation also appears to play an important role, as it has been implicated in protein kinase A (PKA)‐mediated tau phosphorylation, suggesting that it might be an early event that induces further hyperphosphorylation.^[^ ^29^ ^]^ The presence of negatively charged amino acids in tau, obtained after hyperphosphorylation, induces tau folding.^[^ ^30^, ^31^ ^]^ This conformation modification of tau is closely associated with an initial stage of tau aggregation, followed by cleavage at aspartic acid^421^, believed to occur before the appearance of a late conformational modification and truncation at glutamic acid^391^.^[^ ^30^, ^32^, ^33^, ^34^ ^]^ These findings indicate that tau phosphorylation triggers the pathological pathway and represents a pivotal posttranslational modification in the early stages of AD.

### 2.3 Enzymes related to tau hyperphosphorylation

Phosphorylation of tau occurs through the activity of tau kinases like protein kinase A (PKA), protein kinase C (PKC), mitogen‐activated protein kinases (MAPKs), proline‐directed protein kinases, such as extracellular signal‐regulated kinase (ERK), glycogen synthase kinase 3 beta (GSK‐3β), casein kinase 1 epsilon (CK1ε), and cyclin‐dependent kinase 5 (CDK5), which target S/T residues in S/T‐P sequences.^[^ ^35^, ^36^, ^37^ ^]^ Many of the 16 S/T‐P sequences have been identified as irregular phosphorylation sites in AD. Downregulation of protein phosphatases is another mechanism contributing to tau hyperphosphorylation, as these enzymes account for the dephosphorylation of tau. Among these enzymes, protein phosphatase 2A (PP2A) is responsible for approximately 71% of tau phosphatase activity. In contrast, protein phosphatase 1 (PP1), protein phosphatase 5 (PP5), and protein phosphatase 2B (PP2B) represented only approximately 28%.^[^ ^38^, ^39^ ^]^ It is important to emphasize that tau phosphorylation exerts multiple effects at different phosphorylation sites. An in vitro study found that tau phosphorylation at sites S199, S202, T205, T212, S235, S262, and S404 could induce the tau conformation required to sequester normal tau.^[^ ^40^ ^]^ The same study suggested that phosphorylation at sites T231, S396, and S422 is essential for tau self‐assembly, while phosphorylation at either T181 or T217 may regulate this process. Additionally, it has been suggested that phosphorylation at S396 and S404 facilitates the “unfolding” of the C‐terminal, thereby promoting tau assembly.^[^ ^41^ ^]^ Analyzing the sites that affect the binding of tau to microtubules revealed that phosphorylation of S235 induces approximately 9% of the overall binding inhibition. T231 and S262 contributed approximately 26% and 33%, respectively.^[^ ^42^ ^]^ Hence, an efficient approach to treat AD should focus on targeting the initial stage of tau pathology, hyperphosphorylation‐induced aggregation.

## 3 CURRENT THERAPEUTIC STRATEGIES AGAINST TAU PATHOLOGY IN AD

### 3.1 Enzymes modulators

Reducing tau hyperphosphorylation by blocking protein kinases, such as GSK‐3β, CDK5, and c‐Jun N‐terminal kinases (JNK) is one alternative to treating AD, as their levels have been found to increase in AD brains.^[^ ^43^, ^44^, ^45^ ^]^ Additionally, studies have shown that the downregulation of PP2A can lead to AD‐like pathology, including tau hyperphosphorylation and destabilization of microtubules.^[^ ^46^ ^]^ As such, activating this phosphatase is another strategy against AD.^[^ ^47^ ^]^

### 3.2 Inhibitors of tau polymerization

While it was previously hypothesized that tau fibrils were the leading cause of toxicity in tauopathy models, recent research has proposed that tau oligomers, an intermediate in the formation of tangles, are the actual cause of toxicity in these cases.^[^ ^48^ ^]^ A study of a model of tauopathy with *Drosophila melanogaster* found neurodegeneration without the characteristic NFTs.^[^ ^49^ ^]^ Thus, inhibiting the aggregation of tau is crucial for reducing toxicity and neurodegeneration in AD. For example, methylene blue proved effective against tau aggregation in vitro by blocking tau–tau binding.^[^ ^50^ ^]^ However, subsequent clinical trials fail to observe significant positive effects in patients with mild AD.^[^ ^51^ ^]^

### 3.3 Microtubule stabilizers

In AD, tau unbinds from microtubules, destabilizing them and disturbing axonal transport, leading to neurodegeneration. Molecules acting as microtubule stabilizers to prevent disruption have been studied as a possible therapy against AD.^[^ ^52^ ^]^ Among these, paclitaxel, an approved and utilized medicine in treating cancer, has demonstrated efficacy in ameliorating fast axonal transport deficits and increasing microtubule levels. However, its limited ability to penetrate the blood–brain barrier (BBB) renders it unsuitable for treating tauopathies.^[^ ^53^, ^54^ ^]^ Epothilones, on the other hand, are shown to be an alternative to paclitaxel, improving the microtubule levels and its stabilization, as well as axonal integrity, while bearing the capacity to cross the BBB.^[^ ^54^, ^55^, ^56^ ^]^

### 3.4 Immunotherapy

Immunotherapy against Aβ has demonstrated limited effectiveness with controversial results in numerous clinical trials, often accompanied by severe side effects. Consequently, research efforts have focused on active and passive immunization against tau.^[^ ^57^, ^58^ ^]^ A study on the effects of active immunization on P301L tangle model mice suggests a potential delay in tauopathy progression, particularly beneficial in its early stages.^[^ ^59^ ^]^ Likewise, passive immunization using the PHF1 antibody on homozygous female JNPL3 mice showed a reduction in tauopathy without apparent adverse effects. Nonetheless, the passive immunization approach remained less effective than the active immunization.^[^ ^60^ ^]^ Studies focusing on passive immunization reported decreased tau oligomer levels in P301L mice, ameliorating their motor and cognitive deficits without detrimental effects on health or reduced NFT levels.^[^ ^61^, ^62^ ^]^

### 3.5 Adverse effects associated with the regulation of molecular pathways involved in AD

The safety of using plant extracts or isolated molecules from plants as potential therapies for AD is a critical consideration in their development. Plant‐based compounds are used because of their antioxidant, anti‐inflammatory, and neuroprotective properties. Nonetheless, plant extracts frequently involve a variety of bioactive compounds, which may interact inaccurately with different targets. Potential risks include toxicity at high doses, adverse interactions with other medications, and variability in extract composition. Therefore, rigorous preclinical and clinical studies are required to assess their safety, efficacy, and appropriate dosing. The standardization of extracts and the implementation of regulatory oversight are essential to guarantee their safe application in treating AD. This review focuses on those plant extracts or molecules targeting tau pathology; however, various molecular mechanisms associated with their effects might induce side effects.

Targeting kinases to treat AD has been developed as a therapy; this strategy may reduce inflammation and prevent neuronal death, thereby ensuring neuroprotection. Protein kinase inhibitors offer a potential treatment to address pivotal molecular mechanisms of AD.^[^ ^63^ ^]^ GSK‐3β is highly activated in individuals diagnosed with mild cognitive impairment and AD.^[^ ^64^ ^]^ Different research groups provide substantial evidence regarding the role of GSK‐3β in the implications of AD, including its correlation with the hyperphosphorylation of tau, increased production of Aβ, cognitive deficits, neuroinflammation, and neuronal damage loss.^[^ ^65^ ^]^

Interestingly, while this kinase regulates important physiological molecular mechanisms,^[^ ^63^ ^]^ its inhibition in healthy rodents does not show remarkable effects on behavioral cognitive scores,^[^ ^66^ ^]^ suggesting that GSK‐3β is a safe therapeutic target. Nevertheless, genetic reduction^[^ ^67^ ^]^ or overexpression^[^ ^68^, ^69^ ^]^ of the enzyme induces cognitive impairments; however, the abnormal effect of overexpressed proteins is observed in any transgenic animal that disrupts the balance of the normal protein expression. The potential of GSK‐3β inhibitors to prevent AD‐like pathology in murine models has led to several clinical studies on patients. Therefore, contradictory data concerning the efficacy of these treatments have been reported across different clinical trials.^[^ ^70^ ^]^ GSK‐3 is essential for cell survival, raising concerns that its inhibition could result in cellular impairments.^[^ ^63^, ^71^ ^]^ Consequently, only a limited number of GSK‐3β inhibitors have advanced from preclinical to clinical stages. In preclinical studies, tideglusib has been reported to reduce tau phosphorylation, Aβ deposition, neuronal loss, and gliosis in the mouse entorhinal cortex and hippocampus and reverse spatial memory deficits in transgenic mice.^[^ ^72^, ^73^, ^74^ ^]^ In the clinical phase, tideglusib demonstrated favorable tolerability, providing valuable safety and efficacy, except for a transient elevation in serum transaminase levels. Although insufficient evidence supports or rejects the benefit of tideglusib in AD,^[^ ^70^ ^]^ regulating GSK‐3β activity seems promising for treating AD because of its safety and few side effects.

CDK5 is another kinase that regulates tau. It is essential for developing the central nervous system, differentiation, synaptic functions, and memory consolidation. However, during pathological conditions, the complex cdk5‐p25 will over‐activate calpain, ultimately leading to aberrant hyperphosphorylation of various substrates, including tau, amyloid precursor protein (APP), and neurofilament. This dysregulation is implicated in the pathogenesis of neurodegenerative diseases such as AD.^[^ ^75^, ^76^ ^]^

Consequently, protocols to reduce CDK5 activity signify potential therapeutic interventions to reduce or completely inhibit disease progression. Numerous CDK5 inhibitors have been reported in the patent literature.^[^ ^77^ ^]^ A subset of these inhibitors appears to act as ATP competitors; the ATP binding site of CDK5 exhibits high homology with that of other CDK and GSK‐3β.^[^ ^75^ ^]^ Achieving selectivity over these kinases presents an additional challenge in the quest for effective CDK5 inhibitors. Indeed, CDK2 displays a 60% sequence identity with CDK5; it is involved in the physiological regulation of the cell cycle and is considered an anticancer agent,^[^ ^75^ ^]^ given that its inhibition is a potential risk. Therefore, the specific inhibition of CDK5 may present potential adverse effects. Alongside tau, CDK5 is responsible for the phosphorylation of various substrates involved in crucial cellular processes, such as dopamine signaling pathways,^[^ ^78^ ^]^ neurite outgrowth,^[^ ^79^ ^]^ insulin exocytosis,^[^ ^80^ ^]^ and neurotransmitter release.^[^ ^81^, ^82^ ^]^

For instance, some CDK5 inhibitors also display potent inhibitory effects on GSK‐3β.^[^ ^83^ ^]^ The potential lack of selectivity toward this kinase may, in some cases, be advantageous, as compounds that inhibit both enzymes could offer enhanced efficacy.^[^ ^82^ ^]^ Moreover, in vivo and clinical studies required titration to reduce potential collateral effects.

Protein aggregation is a common pathological marker for diseases, such as Parkinson's, Huntington's, and AD. Intracellular tau aggregates are toxic to various cellular processes.^[^ ^84^ ^]^ Likewise, accumulated evidence indicates the importance of tau aggregates in the onset of AD, including the correlation of NFTs with the progression of the disease,^[^ ^85^ ^]^ the tau aggregates spreading AD pathology,^[^ ^86^ ^]^ and the failure of Aβ‐targeting therapies.^[^ ^87^ ^]^ This leads to the hypothesis that preventing aggregate formation, promoting the clearance of tau aggregates, or stabilizing microtubules would be positive in treating these disorders.

Therefore, small molecule inhibitors of protein aggregation are considered promising candidates for AD therapies. Methylene blue chloride (MTC) is recognized as one of the most effective tau aggregation inhibitors assessed in clinical trials. It has been asserted to support the hypothesis regarding tau aggregation inhibition as a potential strategy to treat AD.^[^ ^88^ ^]^ MTC effectively inhibits the tau–tau binding interaction; however, the concentrations required for tau aggregation inhibition may prove toxic to humans when administered orally, as such high levels can result in accumulation within the intestine and liver. Conversely, when administered intravenously, the compound tends to localize and accumulate within the brain.^[^ ^88^ ^]^ Another MTC side effect is the possibility of targeting monoamine oxidases or nitric oxide synthases.^[^ ^89^ ^]^

Considering these studies, it is crucial to emphasize that all evaluated molecules, including those derived from plants, require proper dosing and analysis across various organs to minimize human side effects. Regarding the natural products presented in this review, most of these substances have the potential to block tau fibril formation or facilitate disaggregation. Nonetheless, there is a controversy concerning the significance of this capacity, as prior reports have indicated that NFTs are probably not correlated with functional deficits.^[^ ^84^, ^90^ ^]^ Mice were provided with MTC through their drinking water; this intervention enhanced behavioral outcomes and reduced soluble tau levels without any observed alteration in tau tangle pathology. This suggests that lowering soluble tau levels is beneficial, independent of the contribution from tau aggregates.^[^ ^84^, ^90^ ^]^

Likewise, oligomeric tau may also contribute to the pathogenic mechanism involved in the onset of AD, potentially through extracellular pathways^[^ ^91^, ^92^ ^]^; these hierarchical oligomeric forms of tau could play a role in neurodegeneration. The capability to dissolve preformed aggregates has also been demonstrated with different molecules. However, this often results in the formation of small oligomers, which are hypothesized to cause impairments.^[^ ^93^, ^94^, ^95^, ^96^ ^]^ Thus, tau aggregation inhibitors have shown a moderate degree of success.^[^ ^93^ ^]^ Studies have indicated that tau aggregation inhibitors can prevent the pathology associated with tau, although significant improvements are necessary before this therapeutic approach can be employed.

Using plant‐derived compounds to target molecular pathways involved in AD, such as tau hyperphosphorylation, protein aggregation, and kinase activity, holds significant therapeutic potential. However, toxicity, off‐target effects, and lack of selectivity remain substantial difficulties. Proper dosing, rigorous preclinical studies, and careful analysis of adverse effects are essential for developing safe and effective therapies.

## 4 NATURAL PRODUCTS AGAINST TAU PATHOLOGY IN AD

Numerous research reports on natural products have shown their potential in treating AD pathology. Some of these compounds or mixtures are focused on reducing Aβ‐induced neurodegeneration, with the potential to cross the BBB.^[^ ^97^ ^]^ Another category of interest includes acetylcholinesterase inhibitors,^[^ ^98^ ^]^ which have demonstrated neuroprotective properties, including reducing oxidative stress and enhancing cognition.^[^ ^99^, ^100^ ^]^

Another process modulated by natural products is senescence, a cellular process that involves aging through irreversible growth arrest on cells caused by altered gene expression. Therefore, senescent cells accumulate and contribute to age‐related pathologies. Recent evidence demonstrates that tau protein accumulation induces cellular senescence in the brain.^[^ ^101^ ^]^ Senolytics are synthetic or natural compounds that eliminate senescent cells. Some of the following compounds, obtained from natural products, have demonstrated senolytic properties through different mechanisms and a relatively low toxicity profile in humans. Quercetin, fisetin, apigenin, resveratrol, epigallocatechin, oleuropein, hydroxytyrosol, curcumin, and apigenin belong to natural compounds with senolytic properties.^[^ ^102^, ^103^, ^104^ ^]^

However, given that the toxicity of tau oligomers and NFTs appear to be the best hallmark of AD,^[^ ^24^, ^85^, ^105^ ^]^ preventing their formation could represent a highly effective therapeutic strategy. Hyperphosphorylation‐induced tau aggregates are one of the main events in AD progression.^[^ ^106^, ^107^, ^108^ ^]^ Natural products targeting this tauopathy hold considerable promise in treating the disease while potentially avoiding some side effects associated with current medications.^[^ ^109^ ^]^ Research focusing on natural products and their efficacy against tauopathy is underway, and this review aims to summarize some of the most significant findings in this area.

### 4.1 Modulators of kinase and phosphatase activities

#### 4.1.1 Baicalein

Baicalein, a flavonoid initially extracted from the roots of *Scutellaria baicalensis* Georgi and *Scutellaria lateriflora* L., emerges as one of its primary active constituents. This compound has shown various beneficial properties, including anti‐inflammatory, antioxidant, anticancer, and antiviral properties, highlighting a noticeable medicinal potential.^[^ ^110^ ^]^ Recent studies have focused on baicalein use in neurodegenerative conditions like AD due to its potential against protein misfolding and aggregation. Studies suggested a dual action potential of baicalein, inhibiting tau protein assembly by promoting off‐pathway oligomers and dissolving paired helical filaments in a heparin‐induced tau aggregation model.^[^ ^111^ ^]^ In an in vivo study using AD‐induced mice, baicalein was found to facilitate the recovery of their behavioral deficits.^[^ ^112^ ^]^ Another study employing thioflavin S (ThS) and 1‐anilino‐8‐naphthalene sulfonate (ANS) fluorescence assays demonstrates baicalein's capacity to sequester tau oligomers and stop fibril formation.^[^ ^113^ ^]^ Additionally, it has also been considered that this compound may prevent memory deficits in APP/PS1 mice by inhibiting 12/15 lipoxygenase and GSK‐3β, thereby blocking the hyperphosphorylation of tau.^[^ ^114^ ^]^

#### 4.1.2 Caffeine

Consumption of coffee has been associated with memory and learning improvement and decreased AD symptoms. Different components of coffee have been assessed, including chlorogenic acid, quinic acid, caffeine, quercetin, caffeic acid, and phenylindane. Nevertheless, only a few demonstrated an effect on tau.^[^ ^115^ ^]^ Caffeine, an alkaloid belonging to the xanthine group, is the most widely consumed psychoactive drug. It is primarily extracted from the seeds of the *Coffea* plant genus, native to South Asia and subtropical Africa. A case–control report provided direct evidence that consuming caffeine is associated with diminished dementia risk or with its delayed onset.^[^ ^116^ ^]^ In vitro studies have indicated that caffeine prevents Aβ‐induced neurotoxicity by blocking adenosine receptor A_2A_.^[^ ^117^ ^]^ While most research has focused on its antioxidant property or its effects on preventing Aβ accumulation, there is growing interest in its impact on AD‐like tauopathy models. A study on male THY‐tau22 mice demonstrated that chronic caffeine ingest reduces tau hyperphosphorylation in the hippocampus and diminishes neuroinflammation.^[^ ^118^ ^]^ Another study on white male New Zealand rabbits administered with a high‐cholesterol diet produced increased Aβ, oxidative stress hippocampal levels, and tau hyperphosphorylation; caffeine exhibited regulation of tau phosphorylation.^[^ ^119^ ^]^

#### 4.1.3 Cinnamon

Cinnamon is a spice from the *Cinnamomum* genus collected from the inner bark of the trees, and it owes its flavor and aroma to cinnamaldehyde, the primary component extracted from its essential oil. In vitro studies have revealed potential therapeutic effects of cinnamon. An aqueous extract of *Cinnamomum zeylanicum* inhibited tau aggregation and potentially caused NFT dissociation, possibly by trapping tau monomers or oligomers.^[^ ^120^ ^]^ Another study focusing on the effects of cinnamaldehyde and epicatechin, both active components of cinnamon, demonstrated their ability to protect tau from oxidation and prevent further NFT formation.^[^ ^121^ ^]^ Moreover, the cinnamon extract may diminish tau phosphorylation by modulating the activity of GSK3, potentially through protein kinase A‐dependent phosphorylation inhibition. This effect was demonstrated in an in vitro study with HEK293 cells transfected with GSK3 and tau with cinnamon extract.^[^ ^122^ ^]^ The oral administration of the cinnamon aqueous extract to NTAD rats showed an increase in the inactive form levels of GSK‐3β, phosphorylated at S9, thus less active GSK‐3β, and, therefore, a decrease in tau phosphorylation. Moreover, the extract bears an acetylcholinesterase inhibitor capacity, showing an improved learning ability in NTAD rats.^[^ ^123^ ^]^

#### 4.1.4 Icariin

Icariin, a natural prenylated flavonoid isolated from various species of *Epimedium* genus, commonly known as fairy wings, is predominantly sourced from regions including Asia and the Mediterranean. An in vitro study on PC12 cells showed that icariin inhibits the hyperphosphorylation of tau at the sites S396, S404, and T205. The underlying mechanism of icariin could be attributed to the activation of the PI3K/Akt signaling pathway, inhibiting GSK‐3β and further decreasing tau hyperphosphorylation. Additionally, icariin prevents the cytotoxicity of Aβ_25–35_.^[^ ^124^ ^]^ Further research conducted on SH‐SY5Y cells treated with formaldehyde and icariin doses found that it suppressed hyperphosphorylation of tau at S396 and T181, as well as inhibiting the effects of formaldehyde‐activated GSK‐3β by Y216 phosphorylation.^[^ ^125^ ^]^ In a study with okadaic acid(OA)‐induced tau hyperphosphorylation in the same cell model, treatment with icariin and icaritin proved effective at reducing hyperphosphorylated tau and GSK‐3β levels. Icaritin, a derivative of icariin, exhibited the highest efficacy.^[^ ^126^ ^]^

#### 4.1.5 Luteolin

Luteolin (3′4′,5,7‐tetrahydroxyflavone), a flavone extracted from *Reseda luteola* L. leaves and bark, is a native European and Western Asian plant showing promising therapeutic potential in AD. In an in vivo model for traumatic brain injury, Tg2576 mice demonstrated that daily administration of luteolin reduced AD pathology caused by the trauma, including tau hyperphosphorylation, GSK‐3β activity, and reduced Aβ deposition.^[^ ^127^ ^]^ Furthermore, luteolin exhibited notable effects on SH‐SY5Y cells exposed to high zinc concentrations, an inducer of tau fibrillization in vitro. Luteolin reduced tau hyperphosphorylation at S262 and S356 via control of the phosphorylation/dephosphorylation system and its antioxidant activity.^[^ ^128^ ^]^ In an AD mouse model employing streptozotocin, luteolin‐loaded chitosan decorated nanoparticles demonstrated anti‐inflammatory and antioxidant properties and inhibited tau hyperphosphorylation and Aβ aggregates formation.^[^ ^129^ ^]^ Moreover, luteolin's activity as an acetylcholinesterase inhibitor, among other properties, indicates its therapeutic potential in treating AD.^[^ ^130^, ^131^ ^]^

#### 4.1.6 Dendrobium nobile lindl

Alkaloids‐enriched extract from *Dendrobium nobile* (EDNLA) are compounds, known for their anti‐inflammatory effect, used to treat fever and pain. The EDNL plant is native to southern China, the Himalayas, and Indochina. To investigate the impact of EDLNA in AD, an in vivo study focused on their effect on lipopolysaccharide (LPS)‐induced tau hyperphosphorylation using Sprague−Dawley rats concluded that these alkaloids could decrease the expression of GSK‐3β, thus reducing tau phosphorylation at S199, S202, S396, and S404.^[^ ^132^ ^]^ Furthermore, EDNLA exhibited promising results in aged SAMP8 mice since it effectively diminished cognitive deficits by suppressing the activities of CDK5 and GSK‐3β, thus reducing tau hyperphosphorylation.^[^ ^133^ ^]^ These, along with its properties against the accumulation of Aβ_1–42_ in the hippocampus and its capacity to enhance learning and memory in a mouse model,^[^ ^134^, ^135^ ^]^ DNLA, is proposed as a potential candidate for treating AD. However, further research is required to elucidate its therapeutic mechanisms and fully optimize its clinical application.

#### 4.1.7 Tolfenamic acid

Tolfenamic acid, a nonsteroidal anti‐inflammatory compound categorized as a fenamate and N‐substituted anthranilic acid derivative, is commonly prescribed in Europe for migraine treatment under the brand Clotam®. Studies have highlighted the effects of tolfenamic acid on AD‐related processes. Notably, it has been used to inhibit the protein 1 (Sp1), a transcription factor involved in AD pathogenesis, while reducing the formation of Aβ and tau hyperphosphorylation, thereby improving cognitive functions.^[^ ^136^, ^137^ ^]^ Moreover, after treating APP YAC transgenic mice with tolfenamic acid, the expression of tau and CDK5 was reduced, leading to less phosphorylated tau at sites S235 and T181.^[^ ^138^ ^]^ Although the precise molecular mechanism by which tolfenamic acid induces Sp1 degradation remains unclear, its impact on AD pathology is remarkable. In another study, long‐term memory and cognitive functions were improved in hTau transgenic mice after treatment with tolfenamic acid, associated with a decrease in total and phosphorylated tau levels, mainly at T181 and S396 sites.^[^ ^139^ ^]^ Likewise, tau phosphorylation decreased at S202, T231, and S396 sites in Wistar rats and C57BL/6J mice administered with tolfenamic acid. The effect was attributed to the inhibition of GSK‐3β and increased PP2A activity. Tolfenamic acid also prevented impairments in long‐term memory, working memory, visual recognition, recall memory, and spatial memory.^[^ ^140^ ^]^

#### 4.1.8 Morroniside

Morroniside, a carbocyclic iridoid glycoside isolated from *Cornus officinalis* Siebold & Zucc., belonging to the Cornaceae family, is a Chinese medicinal herb widely utilized in treating various ailments, including dementia and age‐related diseases.^[^ ^141^, ^142^, ^143^ ^]^ Morroniside bears a low toxicity profile and exhibits multiple pharmacological activities ranging from treating rheumatoid arthritis and mitigating vascular complications of diabetes to modulating immune responses and protecting against cerebral ischemia‐reperfusion injury‐induced cell apoptosis while promoting tissue regeneration.^[^ ^144^, ^145^, ^146^, ^147^, ^148^ ^]^ Morroniside's therapeutic potential in AD has gained attention, particularly in targeting the calpain pathway, which plays a key role in AD pathogenesis by regulating kinases and phosphatases. In studies conducted on P301S tau transgenic mice and N2a cells, a synergic effect of morroniside and loganin showed a reduced ratio of 150 kDa/250 kDa spectrin α II, thereby attenuating calpain activity induced by MAPT overexpression. Furthermore, morroniside administration reversed the truncation of spectrin α II stimulated by dibucaine in N2a cells, leading to increased activity of PP2A and decreased GSK‐3β levels. The authors suggest that morroniside reduces tau phosphorylation at S404 and S262 sites through the calpain 1/GSK‐3β/PP2A pathway.^[^ ^149^ ^]^ Moreover, in human neuroblastoma SK‐N‐SH cells pretreated with morroniside, inhibition of OA‐induced damage and restoration of the morphology of cells were observed. Morroniside effectively reduced phosphorylation levels of tau at sites pS199/S202 and pT217 with a concentration of 100 M, while a higher concentration (200 M) further decreased phosphorylated tau at T205, T212, and S214. Morroniside inhibits tau hyperphosphorylation, interacting directly with PP2A and independently of GSK‐3β. Significantly, morroniside did not affect tau phosphorylation in normal SK‐N‐SH cells, highlighting its selective action against pathological processes implicated in AD.^[^ ^147^ ^]^

#### 4.1.9 Salidroside

Salidroside, a phenylpropane glycoside compound extracted from *Rhodiola rosea* L., belongs to the Crassulaceae family and is used in European and Asian folk medicine to prevent or treat different diseases. Its pharmacological benefits include potent efficacy in cerebral ischemia, hypoxia, neurodegenerative diseases, fatigue, diabetes, depression, anxiety, and cancer.^[^ ^150^, ^151^, ^152^, ^153^ ^]^ Interestingly, the research community has considered the potential of salidroside against AD. In vitro studies have revealed the remarkable neuroprotective properties of salidroside; it reduces reactive oxygen species accumulation, attenuates cytotoxicity, decreases intracellular malondialdehyde levels, restores anomalies in the mitochondrial membrane potential, and decreases Aβ levels in an Aβ_1–42_‐primary neurons model.^[^ ^150^ ^]^ Furthermore, studies on tau transgenic Drosophila treated with salidroside have shown promising outcomes, including locomotor activity, longevity, and neuroprotection improvement. This observed efficacy is attributed to the ability of salidroside to reduce tau hyperphosphorylation by regulating the activity of GSK‐3β, a key regulator implicated in AD pathology.^[^ ^154^ ^]^

#### 4.1.10 Asiatic acid

Asiatic acid, a pentacyclic triterpenoid compound extracted from *Centella asiatica* (L.) Urb is commonly used in Chinese and Indian traditional medicine for its remarkable efficacy in enhancing memory and cognitive function. Its pharmacological capacities show efficacy as a potent anti‐inflammatory, antifungal, antioxidant, antiapoptotic, and hepatoprotective.^[^ ^155^, ^156^, ^157^, ^158^ ^]^ In a study performed on undifferentiated PC12 cells, pretreatment with asiatic acid demonstrated significant inhibition of tau hyperphosphorylation induced by Aβ_25–35_ at critical sites, including S396, S404, and T205. Since PI3K/Akt/GSK‐3β signaling pathway is adversely affected by Aβ exposure in AD, the protective effects of asiatic acid were further determined. Notably, the inhibitory effect of asiatic acid on tau hyperphosphorylation was abolished upon administration of LY294002, a specific PI3K inhibitor, suggesting that asiatic acid attenuates Aβ_25–35_‐induced hyperphosphorylation of tau protein through the regulation of the P13K/Akt/GSK‐3β signaling pathway.^[^ ^159^ ^]^ Additionally, Wistar rats were administered with AlCl_3_ and exhibited high levels of tau phosphorylation, leading to neuronal loss, cognitive decline, and high expression of CDK5 in the hippocampus and cortex. Asiatic acid administration decreased the effects of aluminum on inflammation, oxidative stress, CDK5 expression, tau pathology, cognitive impairment, cholinergic deficits, and apoptosis. Therefore, asiatic acid reduces anxiety behavior and improves reference and working memory functions.^[^ ^160^ ^]^

#### 4.1.11 Garlic

Garlic (*Allium sativum* L.) is widely used in gastronomy and traditional medicine as a natural remedy for intestinal disorders, including worms, flatulence, respiratory infections, skin diseases, and wound healing.^[^ ^161^, ^162^, ^163^ ^]^ S‐allylcysteine (SAC) and diallyl disulfide (DADS) are the two primary compounds of aged garlic extract (AGE).^[^ ^163^ ^]^ Studies on spontaneously hypertensive rats (SHR) administered with *Artemisia scoparia* Waldst & Kitam extract, and garlic aqueous extract (ASG) revealed that ASG diets suppressed Aβ accumulation from the brains by enhancing the apolipoprotein E (APOE) receptor LRP1 and reduced the expression of the receptor for glycation end products (RAGE). Furthermore, ASG diets exhibited an inhibitory effect on tau phosphorylation at sites S396 and S404 by suppressing GSK‐3β activity.^[^ ^164^ ^]^ In another study using APP‐Tg mice treated with AGE or SAC, reductions in hyperphosphorylated tau levels were attributed to the decreased activity of GSK‐3β and anti‐amyloid properties induced by garlic compounds. The insulin secretagogue action of garlic compounds increases the brain levels of insulin and insulin‐like growth factor, thereby reducing brain Aβ burden and potentially preventing tau hyperphosphorylation through the inhibition of GSK‐3β activation.^[^ ^165^ ^]^ Further studies on Tg2576 mice administered with AGE, SAC, or DADS corroborated the efficacy of garlic compounds in reducing tau hyperphosphorylation within the central nervous system, with AGE exhibiting the most pronounced effects, followed by SAC and DADS, respectively. These findings emphasize the multitarget neuroprotective properties of garlic compounds.^[^ ^166^ ^]^

#### 4.1.12 Morin and resveratrol

Morin (2′,3,4′,5,7‐pentahydroxyflavone) and resveratrol (3,5,40‐trihydroxy‐trans‐stilbene) are naturally occurring flavonoids and polyphenolic phytostilbenes, commonly found in various plants. Morin can be obtained from guava, apple, osage orange, old fustic, and onion. Resveratrol is abundant in grape skins, peanuts, and notably in red wines. Both compounds exhibit numerous beneficial effects, with morin showing anticancer, anti‐inflammatory, and cardiovascular protective activities. In contrast, resveratrol has potent antioxidative and anti‐inflammatory effects and could act as a powerful anticancer and anti‐diabetes agent.^[^ ^167^, ^168^ ^]^ Different studies have indicated that morin and resveratrol bear the capability to inhibit the aggregation of tau.^[^ ^166^, ^167^, ^168^, ^169^, ^170^, ^171^, ^172^ ^]^ Moreover, in vivo assays have demonstrated their capacity to inhibit the enzyme GSK‐3β or its inhibitory effect on the CDK5 signal pathway, which are implicated in inducing tau hyperphosphorylation, thereby reducing brain levels of phosphorylated tau.^[^ ^170^, ^173^, ^174^, ^175^ ^]^ The potential of morin and resveratrol to prevent tau aggregation and deterioration in motor function was evaluated in transgenic mice overexpressing P301L mutant human tau as a model of frontotemporal dementia. The results showed that resveratrol and morin reduced the levels of hyperphosphorylated tau, observed by immunohistochemistry, although the rotarod test indicated that motor functions remained unaffected.^[^ ^169^ ^]^ In another in vivo study, resveratrol was found to inhibit tau hyperphosphorylation and protect against CdCl2‐induced memory deficits in rat brains by activating the AMPK/PI3K/Akt signaling pathway, thereby simultaneously activating PP2A and inhibiting GSK‐3β. The protective effect of resveratrol on tau hyperphosphorylation was abolished upon coadministration with a selective PI3K inhibitor, LY294002, suggesting the crucial role of the AMPK/PI3K/Akt signaling pathway in its mechanism of action instead of PP2A.^[^ ^176^ ^]^ Additional evidence shows the promising role of resveratrol in the therapy of AD and related tauopathies. This research demonstrated through in vitro and in vivo assays that resveratrol effectively induces a time‐ and dose‐dependent dephosphorylation of tau by increasing PP2A activity and reducing the expression of the ubiquitin ligase MID1.^[^ ^177^ ^]^ Furthermore, it was demonstrated that resveratrol effectively suppresses tau phosphorylation at the S396 site induced by sodium orthovanadate (Na3VO4) in rat brains by downregulating ERK1/2 and GSK‐3β signaling cascades while also reducing oxidative damage.^[^ ^178^ ^]^

#### 4.1.13 Isobavachalcone

Isobavachalcone is a member of the class of chalcones, which is a trans‐chalcone substituted by hydroxy groups. It is also known as coryllifolinin, obtained from the dried ripe fruit of the leguminous plant *Psoralea corylifolia* L. It is known for its pharmacological activities, including neuroprotective, anticancer, antimicrobial, antioxidant, and anti‐inflammatory properties.^[^ ^179^ ^]^ In neurodegenerative diseases, isobavachalcone inhibits tau K18 aggregation and disaggregates tau K18 fibrils, elucidated through ThT fluorescence assays. Biophysical techniques such as biolayer interferometry and HSQC NMR spectroscopy demonstrated the interaction between isobavachalcone and tau K18, revealing specific binding sites at residues N265, I278, S285, C291, K294, I297, V309, V318, and K340. Additionally, the cellular protective capacity of isobavachalcone against tau oligomers was assessed using the CCK‐8 method, demonstrating its ability to prevent apoptosis induced by tau oligomers through mitochondria and endoplasmic reticulum‐mediated apoptotic pathways. Moreover, in vivo experiments revealed the potential of isobavachalcone in reducing tau protein phosphorylation at four sites, T231, S396, S404, and S422, associated with tauopathies impairments, by regulating GSK‐3β and PP2A.^[^ ^180^ ^]^ In another study, isobavachalcone was administered to 3×Tg‐AD mice, which resulted in improved memory, recognition, and anxiety, along with a reduction in the accumulation of Aβ oligomers and tau hyperphosphorylation. Metabolomic analysis revealed that isobavachalcone affects the tyrosine and purine metabolic pathways and bile secretion. These findings indicate that isobavachalcone has excellent potential as a drug candidate for treating AD.^[^ ^181^ ^]^

#### 4.1.14 Grape‐seed extract

The grape seed extract is primarily composed of proanthocyanidin derived from crushed whole grape seeds, a prominent phenolic compound classified within the flavonoid group of plant phytochemicals, specifically belonging to the subgroup of tannins. Its high antioxidant content has been related to various health conditions, including prevention and protection against oxidative stress, tissue damage, and inflammation.^[^ ^182^, ^183^ ^]^ An in vitro study evaluated the inhibitory potential of a select grape seed polyphenol extract on the tau peptide assembly into neurotoxic aggregates. Studies have shown that grape seed polyphenol extract (GSPE) can positively impact tau‐mediated neuropathological responses. When GSPE is combined with synthetic tau peptide Ac‐306VQIVYK311 (at a ratio of 1:10 GSPE:tau peptide), it interferes with the formation of tau aggregates. It can also dissociate pre‐existing tau peptide aggregates in a dose‐dependent manner. These findings suggest that GSPE could be beneficial in preventing the accumulation of neurotoxic tau fibrils in the brain by interfering with their stability.^[^ ^184^ ^]^

The efficacy in preventing AD‐type tau neuropathology was evaluated in vivo.^[^ ^185^ ^]^ The results showed that grape seed extract prevented tau protein aggregation in ordered β‐sheet conformers or protofibrils. Notably, the oral administration of the extracts significantly reduced insoluble tau aggregates in the TMHT mouse brain. Moreover, a significant reduction in tau phosphorylation at critical epitopes T181 and S396/400/404, known to be phosphorylated by ERK 1/2 and GSK‐3β, was observed. Furthermore, oral treatment with the grape seed extracts selectively reduced ERK 1/2 activity without affecting GSK‐3β activity.^[^ ^185^ ^]^ According to a recent study, grape seed proanthocyanidins (GSPs) have been found to possess effective properties that inhibit tau aggregation repeat domain (tau‐RD) and disintegrate preformed and mature tau‐RD filaments in a concentration‐dependent manner. Moreover, the study demonstrates that GSPs can safeguard SH‐SY5Y cells from the damage induced by tau‐RD aggregates. Molecular dynamics simulations reveal that GSPs effectively interact with tau‐RD through hydrogen bonds, hydrophobic interaction, and p–p stacking, which account for their inhibitory activity against tau‐RD aggregation. The authors suggest that GSPs could be a novel treatment to reversibly inhibit AD in patients.^[^ ^186^ ^]^

#### 4.1.15 Rottlerin

Rottlerin is a polyphenol extracted from *Mallotus philippinensis* (Lam.) Müll.Arg. Recently, it was found that rottlerin considerably diminished intracellular phosphorylated and total tau levels throughout autophagy activated by TFEB and Nrf2. The authors suggest that rottlerin might be used as a preventive and therapeutic drug for AD.^[^ ^187^ ^]^

#### 4.1.16 Physalin B

Physalin is a sterane with a high oxidation level commonly found in various plants of the Solanaceae family. This compound exhibits diverse pharmacological activities, such as antitumor, anti‐inflammatory, antibacterial, and antimalarial.^[^ ^188^ ^]^ Physalin B's effect in treating neurodegenerative diseases has rarely been reported. A study performed on HEK293/Tau cells pretreated with Physalin B demonstrates that phosphorylation levels of tau at sites involved in AD onset, like S404, S262, and T231 decreased significantly through the inhibition of GSK‐3β and ERK.^[^ ^189^ ^]^

#### 4.1.17 Punicalagin and ellagic acid

Punicalagin and ellagic acid are polyphenols found in *Punica granatum* L. They are known for their biological activities as antioxidant, anti‐inflammatory, antitumor, and useful in the prevention of cardiovascular and cerebrovascular diseases.^[^ ^190^ ^]^ It has been demonstrated that ellagic acid can ameliorate learning and memory impairments in APP/PS1 mice, significantly reduce neuronal apoptosis and Aβ deposition in the hippocampus, and inhibit the hyperphosphorylation of tau by decreasing the activity of GSK‐3β.^[^ ^191^ ^]^ In another study with H4 line cell, punicalagin and ellagic acid reduced tau phosphorylation at threonine 18; it is suggested that both compounds possess the ability to inhibit the enzyme GSK‐3β, as well as its inhibitory effect on the CDK5 signaling pathway.^[^ ^192^, ^193^ ^]^

#### 4.1.18 Gallic acid

Gallic acid is a polyphenol found in plants, fruits, and teas from traditional Chinese medicine. Its pharmacological benefits include anti‐inflammation, scavenging free radicals, and antioxidant properties. Gallic acid can alleviate cognitive decline in APP/PS1 transgenic mice reducing Aβ_1–42_ aggregation and neurotoxicity.^[^ ^194^ ^]^ The intragastric administration of gallic acid to APP/PS1 mice improved spatial memory by promoting neurogenesis in the hippocampal dentate gyrus and improving synaptic plasticity. Moreover, it reduces the activity of GSK‐3β, leading to decreased tau phosphorylation and reduced Aβ concentration.^[^ ^195^ ^]^

#### 4.1.19 Harmine

Harmine (7‐methoxy‐1‐methyl‐9*H*‐pyrido[3,4‐*b*]indole) is a β‐carboline alkaloid originally isolated from the seeds of *Peganum harmala*. It has a core indole structure and a pyridine ring. Harmine exhibits several pharmacological activities, including antidiabetic, anti‐inflammatory, antitumor, and neuroprotective properties.^[^ ^196^, ^197^, ^198^ ^]^ Derivatives of harmine bear pharmacological effects similar to those of harmine but demonstrate better antitumor activity and lower neurotoxicity.^[^ ^199^ ^]^ A recent report has detailed the design, synthesis, and evaluation of a series of novel harmine derivatives.^[^ ^200^ ^]^ Experimental results indicated that most of these compounds showed strong activity inhibiting GSK‐3β and DYRK1A in vitro, with the derivative ZLQH‐5 emerging as the most effective compound due to its potent inhibitory effects on both targets. The study confirmed ZLQH‐5's capability to cross the BBB and its potential to reduce OA‐induced hyperphosphorylation of tau in the SH‐SY5Y cell model. Molecular docking studies highlighted that ZLQH‐5 could form stable interactions within the ATP binding pocket of GSK‐3β and DYRK1A. Therefore, it can be concluded that ZLQH‐5 warrants further investigation as a promising GSK‐3β and DYRK1A inhibitor for treating AD.^[^ ^200^ ^]^

#### 4.1.20 Acteoside

Acteoside is a phenylpropanoid glycoside formed by binding caffeic acid and hydroxytyrosol to a glucose moiety through ester and glycosidic bonds, respectively. This compound is prevalent in numerous dicotyledonous plants, particularly those belonging to Oleaceae, Bignoniaceae, Verbenaceae, and Labiatae families. Acteoside exhibits a range of pharmacological activities, including anti‐inflammatory effects, hepatoprotection, antioxidant properties, and the regulation of cell apoptosis.^[^ ^201^ ^]^ A recent study was conducted to investigate the impact of acteoside on the levels of Aβ40 and Aβ42 serum, cortex, and hippocampus, along with Aβ‐related scavenging enzymes, phosphorylated GSK‐3β, and hyperphosphorylated tau in the cortex and hippocampus of APP/PS1 mice using western blot analysis techniques. The findings revealed that acteoside significantly enhanced Aβ degradation, inhibited tau hyperphosphorylation, and decreased the activity of GSK‐3β. These results suggest that acteoside effectively inhibits GSK‐3β activity and may improve the pathophysiological and cognitive deficits associated with AD.^[^ ^202^ ^]^

#### 4.1.21 Prenylated flavanone

Prenylated flavanones are phenolic compounds derived from flavonoids, characterized by a flavanone base structure containing one or more prenyl groups (‐CH_2_‐CH=CH_2_) attached. These compounds are primarily found in plants, especially within the Fabaceae family. Prenylated flavanones demonstrate various compelling biological properties, including antibacterial, anticancer, antioxidant, and anti‐inflammatory effects.^[^ ^203^, ^204^, ^205^, ^206^ ^]^

Recent research tested the neuroprotective potential of three prenylated flavanones isolated from Dalea species in a new in vitro preclinical AD model.^[^ ^207^ ^]^ The results indicated that the flavanones: (−)‐(2*S*)‐5,7,2′,4′‐tetrahydroxy‐5′‐(1″,1″‐dimethylallyl)‐8‐prenylflavanone (referred to as 8PP) and (2*S*)‐4′‐hydroxy‐2′‐methoxy‐5′‐(1″,1″‐dimethylallyl)‐8‐prenylpinocembrin (referred to as Me8PP) demonstrated neuroprotective effects against the 3×Tg‐AD astrocyte conditioned medium (ACM), with 8PP showing greater activity than Me8PP. 8PP reduced GSK‐3β‐dependent hyperphosphorylated tau protein levels in neuronal cultures exposed to 3×Tg‐AD ACM. Additionally, this natural compound inhibits GSK‐3β activity in vitro. However, when directly exposed to 3×Tg‐AD neurotoxic astrocytes, 8PP showed toxicity but also reduced the neurotoxicity caused by 3×Tg‐AD ACM. These findings suggest that prenylated flavanone 8PP may be a promising candidate for further investigation as a potential precursor for developing new therapies for AD.^[^ ^207^ ^]^

#### 4.1.22 Ginkgolic acid

Ginkgolic acid (GA) is a phenolic compound found in the *Ginkgo biloba* tree, mainly in seeds and leaves. It belongs to a family of acids with structures similar to salicylic acid, characterized by a long alkyl chain attached to a phenolic core. Ginkgolic acids have shown cytotoxic activity when assayed against several human cancers in various preclinical models, both in vitro and in vivo.^[^ ^208^, ^209^ ^]^ Moreover, its pharmacological activities include antidiabetic, antiviral, anti‐inflammatory, antibacterial, and anti‐fibrotic effects, as well as reno‐ and neuroprotection.^[^ ^210^, ^211^, ^212^, ^213^, ^214^ ^]^ A study found that GA has protective effects against cognitive impairment in a rat model of AD induced by Aβ. GA was shown to reduce neuronal apoptosis and oxidative stress caused by Aβ_1–42_ both in vivo and in vitro. Importantly, GA significantly inhibited tau phosphorylation and blocked the activation of the SUMO‐1/GSK‐3β signaling pathway. These findings suggest that GA improves cognitive function and inhibits tau phosphorylation through the SUMO1/GSK‐3β pathway, indicating its potential as a treatment for preventing and managing AD.^[^ ^215^ ^]^

#### 4.1.23 Eicosanoyl‐5‐hydroxytryptamide

In a rat model of AD,^[^ ^216^, ^217^ ^]^ another constituent derived from coffee, eicosanoid‐5‐hydroxytryptamine (EHT), demonstrated the capability to reduce AD‐like symptoms induced by the overexpression and activation of the cellular PP2A inhibitor protein (I_2_^PP2A^). This effect is achieved by reducing tau hyperphosphorylation, which results from the activation of PP2A, ultimately leading to enhancements in memory function.^[^ ^218^ ^]^

#### 4.1.24 Xanthoceraside

Xanthoceraside, a natural triterpenoid saponin extracted from the husks of the *Xanthoceras sorbifolium* Bunge plant, also known as yellowhorn, has gained considerable interest for its potential therapeutic effects in AD research. In an initial in vivo study, APP‐transgenic mice were administered different xanthoceraside doses, reducing Aβ deposits in their cerebral cortex and hippocampus. Likewise, it prevents tau hyperphosphorylation by inhibiting GSK‐3β activity and reducing tau phosphorylation levels at S396 and S404.^[^ ^219^ ^]^ Further research concluded that xanthoceraside reduced AD symptoms in male SD rats injected with Aβ_1‐42_ by altering gut microbiota composition.^[^ ^220^ ^]^ Another study analyzed the mechanisms of action of xanthoceraside in APP/PS1 transgenic mice. It demonstrated that xanthoceraside activates the BDNF/TrkB/cofilin signaling pathway, reducing tau levels.^[^ ^221^ ^]^ Similar research on the same transgenic mice model revealed increased phosphorylated GSK‐3β S9, reducing tau hyperphosphorylation.^[^ ^222^ ^]^

### 4.2 Natural products regulating kinases and phosphatases: Future therapeutic concerns

Several natural compounds have been investigated for their potential in treating or preventing AD, mainly due to their ability to regulate kinases, phosphatases, and other pathways involved in neurodegeneration. Among the natural products that regulate kinases and phosphatases, caffeine and resveratrol are the most studied compounds with some clinical evidence.

Caffeine has been one of the most extensively studied compounds in AD. Its neuroprotective properties are well documented through epidemiological evidence, where regular caffeine consumption has been associated with a reduced risk of cognitive decline and a delay in AD onset. However, most of these studies have been performed in animal models of AD.^[^ ^223^ ^]^ Caffeine's capacity to inhibit nonselective adenosine receptors,^[^ ^224^ ^]^ reduce Aβ and tau aggregates, and reduce cognitive impairments suggests this molecule is a promising candidate for treating AD,^[^ ^223^ ^]^ even though it has not yet been established as a therapy. Nevertheless, caffeine is the most extensively consumed psychostimulant, the safety of caffeine intake depends on the dose, age, sex, and health condition.^[^ ^225^, ^226^ ^]^ Consumption of caffeine from various sources at levels up to 400 mg daily is considered safe for healthy adults.^[^ ^223^ ^]^ Consequently, moderate and regular coffee consumption may be a beneficial practice to reduce the risk of dementia.

Resveratrol has also entered human clinical trials. A Phase II study demonstrated that resveratrol is safe and well‐tolerated, with its primary metabolites successfully penetrating the BBB to exert effects on the CNS.^[^ ^227^ ^]^ Furthermore, the study suggests that resveratrol may play a role in reducing brain inflammation and potentially decelerating cognitive decline in select participants.^[^ ^227^ ^]^ Although these findings are promising, they remain inconclusive; therefore, further large‐scale studies are needed to ascertain the long‐term efficacy of resveratrol as a treatment for AD.

Several compounds, including tolfenamic acid, baicalein, luteolin, cinnamon extract, garlic extract, grape‐seed extract, EHT, and xanthoceraside, have neuroprotective effects in preclinical studies. They regulate kinase and phosphatase activity, such as GSK‐3β and PP2A, respectively, reducing tau hyperphosphorylation, while luteolin and cinnamon extract also reduce inflammation. Garlic and grape‐seed extracts combat oxidative stress. Other promising compounds like harmine, physalin B, punicalagin, ellagic acid, and asiatic acid demonstrate potential by targeting other kinases such as DYRK1A^[^ ^228^ ^]^ and signaling pathways like MAPK/ERK to reduce oxidative stress, Aβ production, and improve memory in animal models.

Although these compounds have not progressed to clinical trials, their mechanisms, including kinase inhibition, anti‐inflammatory properties, and antioxidant capabilities, are consistent with the pathology of AD, offering potential opportunities for future therapeutic development.

### 4.3 Disaggregation and inhibition of tau aggregation by direct interaction

#### 4.3.1 Quercetin

Quercetin, a flavonoid in coffee and fruits and vegetables such as apples, red onions, and kale, has been investigated for its potential therapeutic effects in AD. In chronic oral administration studies on 3×Tg‐AD mice, quercetin was proposed to prevent tau hyperphosphorylation in the hippocampus and amygdala over the long term, leading to cognitive improvements.^[^ ^229^ ^]^ In another similar study on 3×Tg‐AD mice, quercetin was administered intraperitoneally, and it was found to reverse tau hyperphosphorylation at S202/T205, thereby ameliorating behavioral deficits.^[^ ^230^ ^]^ Quercetin and its derivative, quercetin‐3‐O‐glucuronide, also reduce GSK‐3β activation, decreasing tau hyperphosphorylation in an OA‐induced AD‐like tau pathology model.^[^ ^231^ ^]^ Research on delivering quercetin via plasma exosomes to enhance its bioavailability in the brain showed no significant toxicity in animals, improving quercetin‐blocking tau hyperphosphorylation by inhibiting CDK5.^[^ ^232^ ^]^ Additionally, nano biocatalysts were investigated for their ability to enhance the release of quercetin from onion extract, with quercetin emerging as the most potent compound capable of disassembling NFTs and blocking tau fibrillization by promoting tau conformational stability that reduces aggregation propensity.^[^ ^233^ ^]^ Furthermore, phenylindane, another compound of coffee, was evaluated using an in vitro tau ThS aggregation assay, and a dual inhibition effect on both Aβ and tau fibril formation was found. Phenylindane was demonstrated to be the most potent inhibitor among six different coffee‐tested compounds. The authors suggested that phenylindane may activate antioxidant and neuroinflammatory pathways or alter genes and protein expression to decrease the Aβ and tau pathogenic load. However, the molecular mechanism associated with phenylindane remains uncertain.^[^ ^115^ ^]^

#### 4.3.2 Curcumin

Curcumin is a compound extracted from the *Curcuma longa* L. that belongs to the Zingiberaceae family and is a polyphenol renowned for its natural yellow coloring and culinary use. Furthermore, curcumin has broad beneficial properties, including antitumor, antioxidant, and anti‐inflammatory effects.^[^ ^234^ ^]^ Notably, curcumin has been found to inhibit GSK‐3β activity, which offers neuroprotection against Aβ‐induced mitochondrial metabolic deficiency and atypical alteration of oxidative stress, both affecting tau aggregation.^[^ ^235^ ^]^ An in vivo study with EDNLhTau transgenic mice revealed that curcumin suppressed soluble tau dimer formation and corrected aberrant excitatory synaptic protein expression, indicating its potential for reducing tau fibrillization and providing neuroprotection.^[^ ^236^ ^]^ Interestingly, the biochemical mechanism for these effects was associated with inhibiting β‐sheets formation in tau, an initial step of tau polymerization.^[^ ^237^ ^]^ In another study using a p25 single transgenic mice model, curcumin reduced multiple pathologies, including intraneuronal accumulation of tau and Aβ, by inhibiting CDK5 activity. This inhibition ameliorated neuronal apoptosis and cognitive deficits, highlighting curcumin's potential therapeutic value in neurodegenerative diseases.^[^ ^238^ ^]^

#### 4.3.3 Quercetagitrin

Quercetagetin‐7‐O‐glucoside also known as quercetagitrin, is a flavonol glycoside extracted from cempasúchil or marigold (*Tagetes erecta* L.). This compound has demonstrated antioxidant, anti‐inflammatory, and antihypertensive activity and has been used to treat metabolic disorders.^[^ ^239^, ^240^ ^]^ A transgenic mouse model expressing P301S‐tau showed that quercetagitrin mitigates gliosis, neuronal loss, synaptic impairment, and cognitive deficits. It was also observed to diminish tau hyperphosphorylation at sites T181, S202, T205, T212, S214, and T231 while reducing tau oligomerization and fibrillization in the hippocampus and cortex. The authors propose that the aromatic structure of quercetagitrin interacts with the hydrophobic β‐sheet secondary structure of tau, blocking the fibril formation.^[^ ^241^ ^]^

#### 4.3.4 Rosmarinic acid

Rosmarinic acid, an ester of caffeic acid that contains a phenolic ring, is commonly found in various plants of the Lamiaceae family. This compound exhibits many therapeutic properties, including antibacterial, anticancer, antidiabetic, antinociceptive, antioxidant, and antithrombotic effects. Moreover, in the nervous system, rosmarinic acid has shown efficiency in treating cognitive deficiencies, enhancing memory, and reducing age‐related memory loss,^[^ ^242^ ^]^ as well as anti‐inflammatory, antioxidant, and antiapoptotic effects.^[^ ^243^ ^]^ Rosmarinic acid regulates tau aggregation, preventing β sheet formation, thus avoiding protein fibrillation. This effect is attributed to its capacity to decrease amide regions I and II, essential for pathological tau protein assembly into β sheets. Molecular docking studies explained the binding of rosmarinic acid to the steric zipper in tau, particularly within the ^306^VQIVYK^311^ hexapeptide, thereby inhibiting tau fibrillization and β sheet formation.^[^ ^244^ ^]^ Likewise, in a 7‐day fluorescence polymerization assay, rosmarinic acid reduced tau assembly, forming spherical oligomeric assemblies instead of fibrils. This suggests a delay in fibril formation and the disassembly of pre‐existing fibrils.^[^ ^245^ ^]^

Additionally, a study employing ThT binding assay and electron microscopy measurements demonstrated the capacity of rosmarinic acid to suppress and disassemble the fibrillation of tau. This mechanism is proposed to involve both a direct covalent modification and noncovalent interaction, stabilizing the soluble monomeric form of tau and blocking the progression of oligomer‐induced fibril formation. This property is shared among rosmarinic acid and other polyphenols bearing adjacent phenolic hydroxyl groups.^[^ ^246^ ^]^

#### 4.3.5 Myricetin

Myricetin is a flavonol found mainly in fruits, vegetables, some teas, and red wine, and it shows potent antioxidant activity compared to other flavonoids. Its diverse pharmacological activities include anticancer, antihypertensive, immunomodulatory, anti‐inflammatory, and analgesic activity.^[^ ^247^ ^]^ Myricetin has been extensively studied for its neuroprotective properties, targeting different molecules.^[^ ^248^ ^]^ A study emphasizes myricetin's ability to inhibit the formation of tau fibrils. Molecular dynamics simulations identified its interaction with the VQIVYK hexapeptide region.^[^ ^249^ ^]^ This sequence is essential for its aggregation through cross‐β interactions. Similarly, another study described myricetin's capacity to inhibit heparin‐induced tau filament assembly in vitro, proposing that its inhibition mechanism may involve the formation of soluble oligomeric tau.^[^ ^250^ ^]^ Nevertheless, there is a discordance with another study that described a method to produce co‐factor‐free fibrils using full‐length tau isoforms and mixtures, which is different from the classical method using heparin‐induced tau fibrils. Notably, the structural characteristics of these fibrils differ from those observed in tauopathies patients' brains. Under these conditions, myricetin failed to prevent tau fibril formation; conversely, it enhances tau aggregation.^[^ ^251^ ^]^

#### 4.3.6 Apigenin

Apigenin is a flavonoid found in some vegetables and fruits, such as spinach, celery, dried oregano, onions, and wheat, and its biological activities include anti‐inflammatory, antioxidant, antiapoptotic, and anticancer properties.^[^ ^252^ ^]^ Apigenin bears the ability to cross the BBB, emphasizing its potential to treat neurological conditions such as AD.^[^ ^253^, ^254^, ^255^, ^256^ ^]^ In studies employing ThT fluorescence assay and cell cultures expressing ΔK280 Tau_RD_‐DsRed folding reporter to analyze tau fibrils quantitatively, apigenin demonstrated efficacy in reducing tau aggregation. This compound also shows neuroprotection through different mechanisms, including reduction of oxidative stress and caspase‐1 activity and stimulation of neurite outgrowth.^[^ ^257^ ^]^ Inflammation and reactive nitrogen species, such as NO, promote tau tyrosine residue nitration, thereby promoting aggregation of tau; apigenin is an indirect tau aggregation inhibitor by modulating cytokine expression in inflammatory cells and reducing nitric oxide (NO) from activated glial cells.^[^ ^258^ ^]^

#### 4.3.7 Fisetin

Fisetin, a natural flavonol commonly found in vegetables and fruits, including strawberries, apples, persimmons, onions, cucumber, and grapes, has shown antineoplastic, antioxidant, and anti‐inflammatory properties.^[^ ^259^ ^]^ Likewise, it has demonstrated a positive impact in preclinical models of a variety of neurodegenerative diseases, such as AD, schizophrenia, Parkinson's disease, Huntington's disease, stroke, and traumatic brain injury.^[^ ^260^ ^]^ In nervous system disorders, fisetin shows neuroprotective effects attributed to different molecular pathways, including its antioxidant and anti‐inflammatory capacities. Specifically in AD, fisetin reduces hyperphosphorylated tau and autophagy activation.^[^ ^261^, ^262^ ^]^ The study revealed that fisetin inhibits the aggregation of the tau fragment K18 and induces its depolymerization in vitro, as demonstrated by ThT fluorescence assays and transmission electron microscopy. Moreover, fisetin exhibited a remarkable ability to prevent the formation of tau aggregates in cells. Computational studies revealed a direct interaction between fisetin and tau K18, demonstrating its capacity to inhibit β‐sheet formation by binding to the primary aggregation nucleation site, thereby reducing protein aggregation.^[^ ^263^ ^]^

#### 4.3.8 Tannic acid

Tannic acid, a natural polyphenol found in gallnuts *Quercus infectoria* Olivier and other oak trees, as well as in fresh fruits and coffee, is widely used in traditional Chinese medicine. It has been reported as an antioxidant, antitumor, antimicrobial, and anti‐inflammatory agent.^[^ ^264^ ^]^ Moreover, its neuroprotective activity has been extensively demonstrated by activating different molecular pathways.^[^ ^264^ ^]^ Tannic acid has shown the ability to inhibit the in vitro aggregation of the R3 peptide, the third repeat unit of the tau microtubule‐binding domain. Employing ThS fluorescence assay, electron microscopy, and calorimetry, it was determined that tannic acid strongly binds to R3, as confirmed by molecular docking to elucidate the binding sites. Besides, as observed under electron microscopy, tannic acid inhibits human full‐length tau protein (tau_1–441_) aggregation.^[^ ^265^ ^]^ Subsequently, it was demonstrated that encapsulated tannic acid in a liposome could cross the BBB using a mouse brain endothelial cell model; besides, it reduces tau aggregation in human neuroblastoma cells SK‐N‐SH.^[^ ^266^ ^]^ In a recent study, the effect of tannic acid on tau aggregation was analyzed by ThT fluorescence assays and transmission electron microscopy. Results revealed its inhibitory effect on the fibril formation of wild‐type tau R2 and R3 peptides. Interestingly, the study also investigated the effect of tannic acid on d‐isomerization of the same peptides, showing that with this modification on the fibril morphology, tannic acid diminishes the potency of tau aggregation inhibition.^[^ ^267^ ^]^

#### 4.3.9 Nimbin and salannin

Nimbin and salannin are intermediate and final limonoids, respectively, and compounds found in the fruit of *Azadirachta indica* A. Juss. Limonoids are highly oxidized triterpenes, commonly known as tetranortriterpenoids or meliacins, recognized for their diverse biological activities, including anti‐inflammatory, antibacterial, anticancer, and antifungal properties.^[^ ^268^ ^]^ However, the role of limonoids in the treatment of neurodegenerative diseases has received limited attention. The effect of limonoids on the inhibitory activity of tau aggregation was evaluated; ThS and ANS fluorescence assays revealed that limonoids effectively prevent the formation of tau β‐sheet structures, inhibiting the formation of its aggregates.^[^ ^269^ ^]^ A microscopy study further demonstrated that limonoids block the formation of large tau aggregates, promoting the formation of short, thin, and fragile aggregates. Cell‐based studies suggest that both limonoids were not toxic to HEK293T cells and could overcome oxidative stress. These results strongly suggest the potential of limonoids in reducing tau‐mediated toxicity and overcoming AD.^[^ ^269^ ^]^

#### 4.3.10 Hydroxytyrosol, oleuropein, and oleuropein aglycone

Hydroxytyrosol, oleuropein, and oleuropein aglycone are natural phenolic derivatives and main constituents in the olive tree, its fruits, leaves, olive oil, and olive oil by‐products. Oleuropein and oleuropein aglycone are the most dominant bioactive compounds in olive fruit, representing 14% of the weight of the dried fruit, while hydroxytyrosol is the primary polyphenol in olive oil. Different studies have shown the remarkable biological properties of these three phenolic derivatives, including antioxidant, anti‐inflammatory, antidiabetic, anti‐atherogenic, anticancer, antimicrobial, antiviral, and anti‐Alzheimer's capabilities.^[^ ^270^ ^]^ The inhibitory potential of oleuropein, oleuropein‐aglycone, and hydroxytyrosol on tau aggregation was evaluated on full‐length and mutated P301L tau proteins. Results from light scattering assays indicated that hydroxytyrosol, oleuropein, and oleuropein‐aglycone inhibit aggregation of P301L tau, where oleuropein‐aglycone showed twofold greater efficacy than methylene blue, a standard reference for tau aggregation inhibition. Even more, oleuropein aglycone demonstrated inhibition of fibrillization of full‐length tau at the same concentration. These findings demonstrated the potent capacity of oleuropein aglycone, particularly at low micromolar concentrations, to prevent fibrillogenesis, positioning it as a promising candidate against tau aggregation‐associated pathologies.^[^ ^271^ ^]^ Recent studies have shown that treatment with an olive‐derived extract enriched in hydroxytyrosol and oleuropein prevents a certain degree of proteotoxicity associated with tau aggregation in *Caenorhabditis elegans*. The observed effects were related to the SKN‐1/NRF2 and DAF‐16/FOXO transcription factors and overexpression of HSP‐16.2, as suggested by RNAi tests. Additionally, the extract rich in hydroxytyrosol and oleuropein prevented oxidative stress and delayed Aβ‐induced paralysis by reducing the presence of Aβ aggregates.^[^ ^272^, ^273^ ^]^

#### 4.3.11 Oleocanthal

Oleocanthal (decarboxymethyl ligustroside aglycone) is a natural polyphenolic anti‐inflammatory compound present in certain extra virgin olive oils, originating from the chemical structure of oleuropein. Recent research has exposed its beneficial effects on pathological conditions associated with chronic inflammation, some specific types of cancer, cardiovascular diseases, degenerative joint diseases, and neurodegenerative diseases such as Parkinson's and Alzheimer's.^[^ ^274^, ^275^ ^]^ In vitro and structure‐function studies into oleocanthal's ability to inhibit filament formation using tau isoform T40 and its corresponding MT‐binding region K18 revealed that oleocanthal prevents tau fibrillation. This inhibition occurs by forming a Schiff base‐type adduct with lysine residues in the PHF6 peptide, which blocks its conversion to the β‐folded sheet.^[^ ^276^ ^]^ Subsequent high‐resolution spectrometry studies characterized the reaction profile of oleocanthal with the tau fibrillogenic fragment K18 under pseudophysiological conditions. Researchers identified specific polypeptide residues involved in covalent bonding that block fibril formation.^[^ ^277^ ^]^ The results showed that oleocanthal is covalently bound to K18 through the interaction of the two carbonyl groups of oleocanthal with the 3‐amino group of lysine residues of K18 and the formation of Schiff bases. It was also reported that K18 is susceptible to nonselective covalent modification due to its destructured conformation; consequently, different tau lysine residues react with the two oleocanthal carbonyl groups to form cyclic pyridinium‐type stable adducts.^[^ ^277^ ^]^ Moreover, further studies conducted by the same research group showed that the two aldehyde groups present in the molecule are pivotal for the inhibitory activity of oleocanthal.^[^ ^278^ ^]^

#### 4.3.12 Xanthohumol

Xanthohumol, a prenylated chalconoid found in the female inflorescence of the hop plant (*Humulus lupulus* L.) and beer, exhibits several health benefits. Xanthohumol is under research for its potential biological properties, such as antioxidant, anticancer, anti‐inflammatory, hypoglycemic, and antilipogenic activity.^[^ ^279^, ^280^ ^]^ In a recent study, the ability of xanthohumol to prevent tau K18 fragment aggregation and disaggregate tau K18 fibrils, as well as its cellular protective capacity against tau oligomers, was reported in N2a cells.^[^ ^281^ ^]^ The results from HSQC NMR spectroscopy showed that xanthohumol interacts directly with tau K18 through residues S258, N265, I278, I297, Q336, D345, R349, and I360, and this interaction does not alter the secondary structure of tau K18.^[^ ^281^ ^]^ In addition, western blot results showed the protective effect of xanthohumol in a phospholipid‐induced K18 tau oligomer model that significantly increased the expression level of cleaved caspase‐3 in N2a cells.^[^ ^281^ ^]^ Likewise, the potential therapeutic effects of xanthohumol were tested on murine neuroblastoma N2a cells expressing human Swedish mutant APP (N2a/APP), a well‐defined cellular model for AD. The study discovered that xanthohumol effectively reduced tau hyperphosphorylation via the PP2A and GSK‐3β pathways. Additionally, the study validated the amelioration of tau hyperphosphorylation by xanthohumol on HEK293/Tau cells, another cell line with tau hyperphosphorylation.^[^ ^282^ ^]^ The data suggest the capacity of xanthohumol against fibrillary pathology and cytoprotection in AD.

#### 4.3.13 α‐Asarone

α‐Asarone is a phenylpropanoid isolated from the traditional Chinese herb *Acorus gramineus* Sol. and the plant of the Acoraceae family. Its pharmacological benefits include improving learning and memory, neuroprotective effects, depression, rheumatosis, and osteoporosis.^[^ ^283^, ^284^ ^]^ Studies have focused on the effect of α‐asarone on AD. The intraperitoneal administration of α‐asarone to C57BL/6J mice showed improved learning and memory and regulated the imbalance of Glu and GABA in the hippocampus.^[^ ^285^ ^]^ In another study, the intraperitoneal administration of α‐asarone on APP/PS1 transgenic mice improved cognitive function by inhibiting neuroinflammation and reducing tau hyperphosphorylated.^[^ ^284^ ^]^ Likewise, employing ThT and ANS fluorescence assays demonstrates α‐asarone's capacity to disassemble the pre‐formed tau fibrils to the granular and linear oligomeric intermediates and inhibition of tau fibrillation/aggregation.^[^ ^286^ ^]^

#### 4.3.14 Red ginseng

Red ginseng is a prominent medicinal herb obtained from *Panax ginseng* through a series of steam and drying processes to enhance the pharmacological efficacy of the bioactive substances present in ginseng. It has been used to treat different diseases, including myocardial ischemia, Type II diabetes, depression, cancer, and dementia properties.^[^ ^287^ ^]^ Some active compounds of red ginseng have been identified, like ginsenosides, polysaccharides, organic acids, and nonsaponin water‐soluble glycosides. Their pharmacological effects are related to treating dementia through the regulation of synaptic plasticity and the cholinergic system.^[^ ^288^ ^]^ Red ginseng ethanolic extract and saponins inhibited tau K18 aggregation and promoted the dissociation of tau aggregates elucidated through ThT fluorescence assays.^[^ ^289^ ^]^ In another study, the intraperitoneal administration of polysaccharide‐rich fraction obtained from red ginseng to 3×Tg‐AD mice showed ameliorated deposition and hyperphosphorylation of tau in the brain, also mitigated neurodegeneration and neuroinflammation in the mice. Moreover, it modulated the aggregation and dissociation of tau K18 in HT22 cells.^[^ ^290^ ^]^

#### 4.3.15 Epigallocatechin‐3‐gallate

Epigallocatechin‐3‐gallate (EGCG) is a potent polyphenolic compound isolated from green tea (*Camellia sinensis* (L.) Kuntze), and it is the most abundant and biologically active catechin. Consumption of green tea and EGCG has been considered beneficial for health, mainly attributed to its antioxidant properties in treating cancer, inflammation, metabolic diseases, and cardiovascular and neurodegenerative disorders, such as Parkinson's and Alzheimer's.^[^ ^291^, ^292^ ^]^ In an in vitro study to assess EGCG's efficacy, the results showed the first evidence that it selectively enhances the degradation of hyperphosphorylated tau species in rat primary culture neurons with no adverse effect on cell viability. Remarkably, this effect was not mediated through the Nrf2 pathway; instead, EGCG modulated essential autophagy adaptor proteins NDP52 and p62, promoting the degradation of pathological tau.^[^ ^293^ ^]^ Furthermore, the EGCG's impact on tau aggregation under physiological conditions was evaluated through a cell‐free aggregation assay using the H‐K18DK280 tau fragment. The results demonstrated its potent inhibition of tau aggregation at highly substoichiometric concentrations, preventing the formation of β‐sheet‐rich aggregates and maintaining the tau fragment predominantly in an unfolded monomeric state. This suggests a specific interaction between EGCG and an early aggregation intermediate in the amyloid formation cascade.^[^ ^294^ ^]^ In another in vitro study, EGCG showed the ability to inhibit full‐length tau aggregation, dissolve tau fibrils and oligomers, and form adducts with repeat tau domains. The isothermal titration calorimetry results suggested that the binding of EGCG to full‐length tau is spontaneous and favorable and occurs in a multi‐site binding event. It is predicted that EGCG does not allow the formation of mature aggregates by degradation of the higher‐order structures it forms. Aggregate‐mediated toxicity in neuroblastoma cells was also found to be rescued by the compound. Molecular docking studies revealed that EGCG interacts with tau primarily through hydrogen bonding and hydrophobic interactions with crucial amino acids, notably K267.^[^ ^295^ ^]^ These findings underscore EGCG's remarkable potential as a therapeutic agent against tau pathology.

#### 4.3.16 Lupeol

Lupeol, also known as clerodol, farganasterol, and monogynol B, is a member of the pentacyclic triterpene family, which includes secondary metabolites widely found in the plant kingdom. Lupeol is present in various plants, fruits, and vegetables, such as mangoes, grapes, aloe vera, tamarind, and olives. Its structure is based on a lupane skeleton, consisting of five fused rings and a hydroxyl group at the C‐3 position. This unique configuration contributes to its chemical stability and bioactivity, offering significant pharmacological properties, including antitumor, antioxidant, anti‐inflammatory, cardioprotective, and antidiabetic effects.^[^ ^296^, ^297^, ^298^, ^299^ ^]^ A recent study evaluated the anti‐inflammatory and anti‐Alzheimer's activity of lupeol in a LPS‐injected mouse model.^[^ ^300^ ^]^ The results showed that LPS treatment increased the levels of inflammatory markers, such as NFκB, TNF‐α, and IL‐1β. Treatment with lupeol reversed the LPS‐induced increases in Aβ, APP, BACE‐1, and p‐Tau in the hippocampus, demonstrating its anti‐Alzheimer properties. Additionally, lupeol inhibited the effects of LPS on pre‐synaptic markers SNAP‐23 and SYP, and post‐synaptic marker PSD‐95.^[^ ^300^ ^]^

#### 4.3.17 Cyanidin

Cyanidin is a compound that is commonly found in a variety of fruits, vegetables, and flowers. It belongs to the anthocyanidin family, which comprises plant pigments that produce red, purple, and blue colors for foods and plants. As a water‐soluble molecule, cyanidin features a fundamental flavonoid structure that includes a flavylium cation group in its core, contributing to its antioxidant and coloring properties. Studies have shown that cyanidin exhibits several neuroprotective effects relevant to AD, such as antioxidant, antiapoptotic, and anti‐inflammatory properties, as well as the inhibition of acetylcholinesterase and butyrylcholinesterase activities, among other beneficial effects.^[^ ^301^ ^]^ Cyanidin promotes nerve growth and differentiation, as demonstrated in a PC12 cell model, to assess its neuroprotective properties against Bisphenol A (BPA)‐induced AD pathology.^[^ ^302^ ^]^ The findings suggest that cyanidin may activate nerve growth factors, promote neuronal differentiation, and reduce tau hyperphosphorylation. This occurs by restoring the Wnt/β‐catenin signaling pathway, contributing to its neuroprotective potential against BPA‐induced AD pathology.

### 4.4 Natural products regulating tau aggregation: Future therapeutic concerns

In the search for effective therapies against AD, targeting tau aggregation has emerged as a promising strategy, given the pivotal role of tau pathology in neurodegeneration. None of the compounds listed in the anti‐aggregatory section have been approved as therapies for AD, at least not evaluated as tau aggregation inhibitors. Still, many of them have shown significant potential in preclinical and early‐phase studies.

Curcumin has attracted substantial attention as a tau aggregation inhibitor. It interacts with the beta‐sheet structure of tau fibrils, effectively preventing their aggregation and disrupting pre‐formed tau tangles. However, the therapeutic potential of curcumin is hindered by poor bioavailability^[^ ^303^ ^]^ and limited capacity to penetrate the BBB.^[^ ^304^ ^]^ Only a few clinical studies have examined curcumin's effect on human cognitive functioning. The results of these studies are inconsistent, some studies report that curcumin has no cognitive improvement effects^[^ ^305^, ^306^ ^]^; in contrast, other studies indicate a positive impact of curcumin on cognition.^[^ ^307^, ^308^, ^309^ ^]^

Furthermore, although research indicates that elevated dosages of curcumin may result in moderate adverse effects in elderly patients, it demonstrates a more favorable safety profile compared to existing pharmacological treatments. Thus, considering that current therapies for cognitive impairment are insufficient and frequently associated with substantial side effects, curcumin emerges as a promising alternative. Interestingly, clinical trials assessing the role of curcumin in AD treatment have focused more on its anti‐Aβ and neuroprotective properties rather than direct tau inhibition.^[^ ^310^ ^]^ However, ongoing research is addressing formulation challenges to enhance its efficacy.

EGCG, a major catechin in green tea, has shown great potential as a tau aggregation inhibitor. EGCG binds to tau monomers, preventing their self‐assembly into toxic oligomers and fibrils. Preclinical studies highlight its ability to protect neurons and improve cognitive function in AD models. Despite these encouraging results, clinical trials using EGCG show poor bioavailability^[^ ^311^, ^312^ ^]^ and multiple side effects, including anxiolytic and hypoglycemic activity, hypochromic anemia, and liver and kidney failure.^[^ ^313^ ^]^ Regarding brain pathologies, Down syndrome patients treated with ECGC and cognitive training performed significantly better on memory tests.^[^ ^314^, ^315^ ^]^ However, ECGC has primarily been examined for its effects on Aβ rather than tau pathology. Although no clinical evidence confirms its anti‐tau effects in humans, EGCG is a promising candidate for therapies targeting tau due to its findings from preclinical studies and diverse neuroprotective properties.

Compounds like quercetin, oleuropein aglycone, oleocanthal, myricetin, rosmarinic acid, and tannic acid prevent tau phosphorylation, misfolding, and fibril formation. Their transition from preclinical studies to clinical therapy has faced significant challenges. Poor bioavailability, limited BBB penetration, and a lack of targeted delivery mechanisms have obstructed their therapeutic application. Efforts to overcome these limitations include developing nanoparticle formulations, liposomal delivery systems, and chemical modifications to improve these compounds’ stability and brain uptake.^[^ ^316^ ^]^

As of the present date, no tau aggregation inhibitors, either natural or synthetic, have received approval as therapeutic agents for AD. Current clinical trials predominantly focus on Aβ plaques, leaving tau pathology relatively underexplored. Nevertheless, the increasing research on tau's pivotal role in neurodegeneration drives new interest in tau‐targeted therapies. Compounds like curcumin, EGCG, quercetin, and oleuropein lead this research, providing a multi‐targeted therapeutic approach that integrates tau aggregation inhibition with anti‐inflammatory and antioxidant properties.

## 5 CONCLUSION

Currently, there are no available treatments to cure neurodegenerative disorders like AD; therefore, the use of natural products emerges as an alternative. While natural products alone may not provide a definitive cure, they bear the capacity to regulate, compensate, and activate different molecular mechanisms for managing the condition and potentially slowing the progression of the disease. Natural products contribute to treating AD due to their bioactive compound content, including polyphenols, alkaloids, and flavonoids, which exhibit anti‐inflammatory and antioxidant properties. These compounds have demonstrated the ability to protect neurons from inflammation, oxidative stress, and cell death while enhancing memory and reducing cognitive impairments. The accumulation of toxic proteins like tau forming NFTs in the brain correlates with AD. At the early stage of the disease, the activation of kinases and downregulation of phosphatases induce tau phosphorylation at multiple sites. Hyperphosphorylation has been shown to induce tau aggregation, which is pivotal in tau pathology. Therefore, natural products have been demonstrated to stop or delay this pathological alteration of tau. In this review, we summarized the most relevant molecules and extracts obtained from natural products with the capacity to regulate tau phosphorylation; these include tolfenamic acid, morroniside, salidroside, asiatic acid, garlic, caffeine, eicosanoyl‐5‐hydroxytryptamide, luteolin, alkaloids enriched extract from *Dendrobium nobile*, icariin, xanthoceraside, morin, and resveratrol. Additionally, the following group of molecules, besides reducing tau hyperphosphorylation, regulates its fibril formation; these are curcumin, baicalein, quercetin, phenylindan, cinnamon extracts, quercetagitrin, nimbin, fisetin, salannin, hydroxytyrosol, oleuropein, oleuropein aglycone, oleocanthal, epigallocatechin‐3‐gallate, xanthohumol, isobavachalcone, rosmarinic acid, myricetin, apigenin, tannic acid, and grape‐seed extract. Table 1 presents the model used, doses/administration, and effects obtained from these extracts or molecules.

**Table 1:** Summary of the models, doses/administrations, and effects obtained from these extracts or molecules.

Extract or molecule	Origin	Model^a^	Administration^b^	Effect on tau
Baicalein	*Scutellaria baicalensis Georgi*	AlCl_3_‐induced AD male Swiss albino Wistar rats	Dosage of 5 and 10 mg/kg/day. Duration: 12 weeks.	Inhibits 12/15LO, GSK3, and secretase enzyme (BACE1) activities.
Caffeine	*Coffea* plant	THY‐Tau22 male mice	Dosage of 0.3 g/L through drinking water. Duration: 12 months.	Reduces tau hyperphosphorylation. Increases Erk phosphorylation and CDK5 expression.
Cinnamon extract	Cinnamon	Thioflavin T fluorescence‐ polymerization assay, using tau‐187	Evaluated concentration over tau aggregation: 110 μM or 22 mg/mL. Duration: 15 h.	Suppresses the GSK‐3β activation and reduces tau hyperphosphorylation.
Luteolin	*Reseda luteola*	Traumatic brain injury and amyloid β depositing Tg2576 mice	Treatment at 20 mg/kg ip. Duration: 15 days.	Reduces GSK‐3β expression and tau hyperphosphorylation.
EDNLA	*Dendrobium Nobile Lindl*.	Lipopolysaccharide‐induced tauopathy Sprague−Dawley rats	Intragastrical administration at 20 or 40 mg/kg. Duration: 7 days.	Reduces GSK‐3β expression and tau hyperphosphorylation.
Tolfenamic acid	*Fenamates*	Male APP YAC transgenic mice Female and male	Oral administration. Dosage: 0.5 or 50 mg/kg/day. Duration: 34 days.	Reduces tau hyperphosphorylation by inhibition of CDK5.
		Tg(MAPT)8cPdav/J transgenic mice	Oral administration. Dosage: 5 and 50 mg/kg/day. Duration: 34 days.	
		Okadaic acid‐induced PP2A inhibition in Wistar rats	Oral administration. Dosage: 10 and 50 mg/kg. Duration: 25 days.	
		PC12 cells	Grown in the presence of 5 and 10 µM. Duration: 24 h.	
Morroniside	*Cornus officinalis* Siebold & Zucc.	P301S tau transgenic mice	Oral administration. Dosage: 50–200 mg/kg/day of morroniside and loganin. Duration: 3 months.	Reduces tau hyperphosphorylation by inhibition of CDK5.
		N2a cells	Grown in the presence of 94 µg/mL. Duration: 24 h.	
		Male Wistar rats	Intragastrical administration at 30–270 mg/kg/day of a mixture of morroniside. Duration: 3 days.	
Salidroside	*Rhodiola rosea L*.	AD transgenic male Drosophila flies	Diet with different concentrations: 2–20 μM Duration: 10–40 days.	Reduces tau hyperphosphorylation by regulating the activity of GSK3β.
Asiatic acid	*Centella asiática* (L.) Urb.	PC12 cells AlCl_3_‐AD model in male albino Wistar rats	Pretreatment with 5–20 µM. Duration: 1 h. Oral administration. Dosage: 75 mg/kg/day. Duration: 6 weeks.	Reduces tau hyperphosphorylation by regulating P13K/Akt/GSK‐3β signaling.
Garlic extract	*Allium sativum L*.	Male spontaneously hypertensive rats	Diet supplemented with 2% (wt/wt). Duration: 6 weeks.	Reduces tau hyperphosphorylation by regulating GSK3β activity.
Morin	*Moraceae family*	ELISA 3×Tg‐AD mice	β‐catenin primed by CKI was incubated in GSH‐coated well plates and active GSK3β, evaluating morin concentrations: 1–5 mM. Administrated once daily i.p. at 10 mg/kg body weight for 7 days.	Inhibits hyperphosphorylated tau by reducing GSK‐3β.
Resveratrol	Grape skins, peanuts, and red wines	JNPL3 tau transgenic mice	Oral administration: 0.1 mg/g/day mixed with peanut butter. Duration: 3 months.	Inhibits hyperphosphorylated tau by reducing GSK‐3β.
Isobavachalcone	*Psoralea corylifolia* Linn	Thioflavin T fluorescence‐ polymerization assay, using recombinant Tau K18	Evaluated concentration over tau aggregation: 0–50 μM. Duration: 24 h.	Inhibits tau aggregation and promotes disaggregation of tau filaments.
		HEK293‐tau cells	Grown in the presence of 4 μM.	Reduces tau hyperphosphorylation by regulating GSK3β and PP2A.
Grape‐seed extract	*Vitis vinifera*	Thioflavin S fluorescence‐ polymerization assay, using Ac‐^306^VQIVYK^311^ tau peptide	Evaluated concentration over tau aggregation: 0.22–22 μM. Duration: 10 min.	Inhibits tau aggregation and promotes disaggregation of tau filaments.
		TMHT mice	Mice were treated with 200 mg/kg/day GSPE, starting at 3 months of age and continued for 2 months.	Interferes with the assembly of tau peptides and mechanisms associated with attenuation of extracellular signal‐receptor kinase 1/2 signaling in the brain.
Rottlerin	*Mallotus philippinensis*	Immortalized mouse cortical neurons that inducibly express full‐length tau Human‐induced pluripotent stem cell‐derived neurons	Treatment with 5 µM. Duration: 12 h. Treatment with 5 µM. Duration: 12 h.	Inhibits GSK‐3β. Inhibits mTORC1 by activating AMPK.
Physalin B	*Physalis* plants	HEK293/Tau cells	Treatment with 1, 2, and 4 µmol L^−1^. Duration: 24 h.	Inhibits GSK‐3β and ERK.
Punicalagin and ellagic acid	*Punica granatum* L.	APP/PS1 transgenic mice H4 and HMC3 cell lines.	Oral administration. Dosage: 50 mg/kg/day. Duration: 60 days. Cells treated with 50 µM. Duration: 24 h.	Decreases the activity of GSK3β.
Gallic acid	Found in plants of traditional Chinese medicine.	APP/PS1 mice	Oral administration. Dosage: 20 mg/kg/day. Duration: 4 months.	Reduces GSK‐3β activity.
Harmine	*Peganum harmala*	ADP‐Glo kinase assay kit	Evaluated concentration over tau aggregation: 500 nM. Duration: 30 min.	Inhibits GSK‐3β and DYRK1A.
Acteoside	Found in plants of the families Oleaceae, Bignoniaceae, Verbenaceae, and Labiatae	Western blot of cortex and hippocampus of APP/PS1 mice samples	Oral administration. Dosage: 50 and 100 mg/kg/day. Duration: 8 weeks.	Inhibits GSK3β activity.
Prenylated Flavanone	Found in plants of the family Fabaceae family	GSK‐3β Kinase Enzyme System and Kinase‐Glo™ luminescent kinase kit	Evaluated concentration aggregation: 1–200 µM. Duration: 30 min.	Inhibits GSK‐3β activity.
Prenylated Flavanone	Found in plants of the family Fabaceae family	GSK‐3β Kinase Enzyme System and Kinase‐Glo™ luminescent kinase kit	Evaluated concentration aggregation: 1–200 µM. Duration: 30 min.	Inhibits GSK‐3β activity.
Ginkgolic acid	*Ginkgo biloba*	Immunohistochemistry and western blot analysis of 10 µg Aβ1‐42 injected mice samples	Evaluated concentration: 30 µg/kg once a day via intracerebroventricular cannulation. Duration: 20 days.	Regulates SUMO‐1/GSK3β pathway.
Eicosanoyl‐5‐hydroxytryptamide (EHT)	*Coffea* plant	Sporadic AD, AAV1‐I_2_‐N/C‐infected Wistar rats	Formulated diet of 0.1% (wt/wt). Duration: 6 and 12 months.	Increases PP2A activity. Reduces tau hyperphosphorylation.
Xanthoceraside	*Xanthoceras sorbifolium*	APP transgenic mice expressing human APP^69K595N/M596L^ of the Swedish mutation	Oral administration of 0.02–0.32 mg/kg/day. Duration: 6 months.	Reduces tau hyperphosphorylation. Increases expression of PP2A and PP1.
Quercetin	Onion skins	Homozygous 3×Tg‐AD mice	Intraperitoneal injections of 25 mg/kg every 48 h. Duration: 3 months.	Reduces levels of paired helical filaments.
Curcumin	*Curcuma longa*	hTau transgenic mice	A diet with 500 ppm. Duration: 5 months.	Reduces soluble tau oligomer formation.
Quercetagitrin	*Tagetes erecta* L	Thioflavin T fluorescence‐ polymerization assay, using tau‐K18	Evaluated concentration over tau aggregation: 3.12–100 μM. Duration: 48 h.	Interacts with tau's hydrophobic β‐sheet secondary structure, blocking the formation of fibrils.
		P301S‐tau transgenic mouse model	Drinking water containing 10 µg/mL. Duration: 3 months.	Restores cognitive deficits and neuron loss in the mice.
Rosmarinic acid	Lamiaceae family	Thioflavin T fluorescence‐ polymerization assay	Evaluated concentration over tau aggregation: 10–100 μM. Duration: 24–168 h.	Inhibits tau aggregation by avoiding fibril formation.
		Molecular Docking. GLIDE docking software		Inhibits tau fibrillization binding to the steric zipper and blocking β sheet formation.
Myricetin	Myricaceae, Anacardiaceae, Polygonaceae, Pinaceae, Primulaceae families, and some vegetables, fruits, and tea.	Thioflavin S fluorescence‐ polymerization assay	Evaluated concentration over tau aggregation: 20 nM to 200 μM. Duration: 72 h.	Induces soluble oligomeric tau inhibiting assembly of tau filaments.
		AMBER11 package molecular dynamic simulation		
Apigenin	*Artemisia family* and from fruits, vegetables, nuts, and herbs	Thioflavin T fluorescence‐ polymerization assay using bacterially expressed proaggregator ΔK280 Tau_RD_	Evaluated concentration over tau aggregation: 5–10 μM. Duration: 48 h.	Inhibits tau aggregation, reduces oxidative stress and caspase‐1 activity as well as promotes neurite outgrowth.
Fisetin	Found in vegetables and fruits	Thioflavin T fluorescence‐ polymerization assay, using tau K18	Evaluated concentration over tau aggregation: 0–186.7 μM. Duration: 15 h.	Inhibits aggregation and depolymerization of the tau fragment K18.
Tannic acid	*Quercus infectoria*	Thioflavin S and thioflavin T fluorescence‐polymerization assays, using R2 and R3 Tau peptides	Evaluated concentration over tau aggregation: 1–25 μM. Duration: 18 h (Tau R3 peptides), 48 h (Tau R2 peptides).	Inhibits tau aggregation by strong binding to peptide R3, suppressing the conformational transition in filament formation. Inhibits tau aggregation through specific binding sites.
		Molecular Docking. Autodock vina		
Nimbin and Salannin	*Azadirachta indica*	Thioflavin S fluorescence‐ polymerization assay, using hTau40 wt	Evaluated concentration over tau aggregation: 400 μM. Duration: 120 h.	Prevents cross‐sheet formation.
Hydroxytyrosol, Oleuropein and Oleuropein aglycone	*Olea europaea L*.	Thioflavin S fluorescence‐ polymerization assay, using Tau‐441 and Tau‐ P301L	Evaluated concentration over tau aggregation: 10 μM. Duration: 16 h.	Inhibits tau aggregation by forming stable covalent adducts with lysine residues.
Oleocanthal	*Olea europaea L*.	Thioflavin S fluorescence‐ polymerization assay, using Tau‐441	Evaluated concentration over tau aggregation: 5–200 μM. Duration: 1 h.	Inhibits tau fibrillation by forming a Schiff base‐type adduct with lysine residues in the PHF6 peptide.
Xanthohumol	*Humulus lupulus* L	Thioflavin T fluorescence‐ polymerization assay, using recombinant Tau K18	Evaluated concentration over tau aggregation: 0–50 μM. Duration: 24 h.	Inhibits tau aggregation and promotes disaggregation of tau filaments by direct interaction with residues S258, N265, I278, I297, Q336, D345, R349, and I360.
α‐Asarone	*Acorus gramineus* Sol.	APP/PS1 transgenic mice Thioflavin T and ANS fluorescence‐polymerization assay	Intraperitoneal injection (single dose) of 30 and 60 mg/kg. Evaluated concentration over tau aggregation: 400 µM, 1, 2, and 10 mM. Duration: 24 h.	Inhibits the aggregation p‐tau Prevents tau fibrillation, disassembles pre‐formed tau fibrils, and disaggregates aggregates.
Korean red ginseng polysaccharide	*Panax ginseng*	3×Tg‐AD mice HT22 cell line. Thioflavin T fluorescence‐ polymerization assay	Oral administration. Dosage: 200 mg/kg/day. Duration: 3 weeks. Cells treated with 100, 250, and 500 µg/mL. Duration: 4 h. Evaluated concentration over tau aggregation: 10, 50, 100, 250, and 500 µg/mL. Duration: 21 h.	Ameliorates deposition and hyperphosphorylation of tau. Modulates aggregation and dissociation of tau K18 in vitro. Inhibits tau aggregation and promotes dissociation of tau aggregates.
Epigallocatechin‐3‐gallate	*Camellia sinensis*	Human SH‐SY5Y neuroblastoma cells	Cells treated with 25 or 50 μM.	Enhances the degradation of hyperphosphorylated tau species.
Lupeol	Found in various plants, fruits, and vegetables	Western blot of lipopolysaccharide‐injected C57BL/6N male mice samples	Oral administration. Dosage: 50 mg/kg/day. Duration: 7 days. Evaluated concentration: 50 mg/kg orally. Duration: 7 days.	Inhibits tau aggregation. Inhibits neuroinflammatory processes.
Cyanidin	*Musa acuminata* Colla	Western blot of the neurite outgrowth of PC12 cells assay	Pretreatment with 90 and 10 µg/mL. Duration: 24 h.	Inhibits tau aggregation. Enhances the activation of the Wnt/β‐catenin pathway.

^a^ Animal or cellular model which was administered the natural product to evaluate its effects.

^b^ Amount of natural product and how it was administered to the model, as well as the duration of this administration.

This report also indicates the proposed molecular mechanisms associated with the effects of these molecules. The most prevalent mechanisms involve the regulation of kinases and phosphatases, primarily GSK‐3β and PP2A, respectively. Likewise, some molecules inhibit tau‐β‐sheets formation, an initial step of tau fibrillation, or bind more strongly to filamentous and aged soluble tau than the monomeric protein, blocking tau–tau interactions during fibril formation. Some other molecules show a potent capacity to depolymerize tau aggregates.

Natural products are considered safer, with fewer side effects. They can be combined with current medications as a complementary therapy, offering diverse options that may improve overall quality of life and well‐being. However, we consider that the described molecules in this review offer an advantage over others; this is the possibility to regulate tau phosphorylation‐induced aggregation. Thus, these molecules not only reduce or control some cognitive impairments and behavioral symptoms temporarily but may delay or stop the progression of the disease.

## CONFLICTS OF INTEREST STATEMENT

The authors declare no conflicts of interest.

## Data Availability

Data sharing is not applicable to this article as no new data were created or analyzed in this study.
